# Perspectives of the Development Strategies for a Future Toxicity Testing System in China: Challenges and Opportunities

**DOI:** 10.1289/ehp.1307303

**Published:** 2013-09-01

**Authors:** Bin Zhao

**Affiliations:** State Key Laboratory of Environmental Chemistry and Ecotoxicology, Research Center for Eco-environmental Sciences, Chinese Academy of Sciences, Beijing, China, E-mail: binzhao@rcees.ac.cn

For many years, toxicological studies in animals have been used to determine potential health risks in humans. There are increasing concerns that such an approach is costly in terms of the resources required to conduct the tests and may not provide sufficient information to adequately protect human health. The growing backlog of chemicals yet to be assessed for potential human health hazards has led to calls to develop high throughput, low-cost testing strategies using end points that are more relevant to humans (Hartung 2009).

In 2007, the U.S. National Research Council (NRC) released a landmark report, *Toxicity Testing in the 21st Century: A Vision and a Strategy* (NRC 2007), that envisioned a revolutionary change in the future of toxicity testing. A similar approach has been described by AXLR8 (AXLR8 Consortium 2011), a project funded under the European Commission’s Seventh Framework Programme. These documents from the NRC and AXLR8 Consortium articulate the current effort to increase efficiency and decrease animal use in toxicity testing by transitioning from *in vivo* tests with qualitative end points to *in vitro* assays based on human cells or cell lines that use robotic high throughput screening and mechanistic quantitative parameters. Implementation of such a strategy could foster a new paradigm that enhances human relevance of toxicological studies, thereby allowing regulatory decisions to be based on human rather than animal biology (Hartung 2009; Schmidt 2009).

Research and regulatory agencies in the United States (National Institute of Environmental Health Sciences, U.S. Environmental Protection Agency, and Food and Drug Administration) and Europe (Environment Directorate General, European Commission) have been major proponents of the new paradigm (Birnbaum 2012; Mahadevan et al. 2011). Others (Andersen and Krewski 2009; Seidle and Stephen 2009) have called on the toxicological community to work together to promote a more international acceptance of this approach. Given the deficiencies of the current toxicological testing approach, it seems logical to develop a global consensus on the new paradigm. There are many challenges to such harmonization because each nation’s particular circumstances—ranging from technical limitations to legislative and economic issues—could hinder the implementation and acceptance of the transition (Hartung 2009).

In China, steps have yet to be taken to fully explore and embrace the move to alternative approaches to toxicity testing. Although ancient China, dating back to 5,000 years ago, is one of the earliest cultures to have an understanding about toxic chemicals, the toxicological testing paradigm in China was established long after the scientific discipline of toxicology began to evolve in other parts of the world. Because of this, a gap exists between China’s current approach to toxicological testing and the cutting edge of the field. This difference is best observed in the methods currently used in China to assess toxicological risks of drugs and chemical substances: Animal testing still represents the dominant testing paradigm, and this has been the norm for the past five decades. Switching to *in vitro* testing would be a timely opportunity for a new start, where Chinese researchers could seize the trend and become pioneers and adopters of alternative strategies for a future toxicity testing system.

From this perspective, challenges and opportunities are equally present in Chinese toxicological research. Certainly, any movement toward a new approach to toxicity testing will face substantial obstacles in China. There are two general barriers to developing a new approach to toxicity testing. First, the new approach will require redistribution of resources and a sustained commitment to support a change in regulations already based on animal testing. For example, in the case of exporting raw materials for cosmetic ingredients, Chinese manufacturers are required to provide two sets of results: one from non-animal tests (intended for the foreign market) and one from animal tests [required by the Chinese State Food and Drug Administration (SFDA), which does not accept data from non-animal testing]. Inevitably, regulations like these have markedly impeded any incentive in China to pay greater attention to non-animal toxicity tests. Second, within Chinese academia, there are no systematic research projects that specifically focus on developing fundamental knowledge and principles to support the testing strategies described by the NRC (2007) or the AXLR8 Consortium (2011). In China, existing research concerning alternative approaches to toxicity testing is sporadic and rate limited. Given these circumstances, the move to develop alternative approaches to toxicity testing in China is at a pivotal point. One thing is clear: Maintaining the status quo will undoubtedly impede progress with regard to advances in toxicity testing and will further separate Chinese regulatory efforts from those in other parts of the international community. It makes scientific and economic sense for Chinese scientists and regulators to participate in generating the knowledge needed to develop more efficient and effective toxicological testing approaches.

At present, there are three paths that China could take to address the need for a better toxicity testing approach: *a*) adopt the framework and methods developed elsewhere and become an active participant and contributor in the development of alternative testing approaches; *b*) modify the existing framework to better suit the particular interests and needs within China’s toxicological research community; or *c*) initiate research programs to independently develop a framework for *in vitro* toxicity testing and computational models of pathway perturbations. Regardless of the actual path taken, China’s scientific community would greatly benefit from this bold progress. Obviously, each path has its own implications with regard to the role China will play in the international toxicological community and the global economy.

By embracing the rapidly evolving paradigm concerning toxicity testing, China stands to benefit in many ways. The development of high throughput methodologies may serve as a major stimulant for basic biological research in China—research that is needed to understand the fundamentals of biological systems and how they interact with toxic chemicals. The development of new methods and increased knowledge will help scientists formulate hypotheses for future research. In addition, there are economic advantages to collaborating with the international scientific community to develop alternative testing strategies; namely, there is a sizable demand for toxicity testing in the global market, especially from the cosmetics and pharmaceutical industries. Because of the incompatible regulations for toxicity testing noted above, Chinese producers, who currently incur high costs for multiple risk assessments for exports, are at a disadvantage that renders them less competitive internationally. Thus, Chinese regulatory agencies should consider the conventional non-animal testing protocols practiced in foreign markets, such that redundancies are eliminated; this would result in an increased appeal to foreign buyers.

If international harmonization of testing approaches is to be achieved, research from China and other countries will be required to incorporate the evolving framework proposed by the NRC (2007) or the AXLR8 Consortium (2011). Chinese researchers can, and should, seize this timely opportunity to make significant contributions to this revolutionary transition in toxicity testing. However, efforts from researchers will not yield meaningful outcomes without full support from regulatory and funding agencies. Policies need to be put in place that recognize the significance of the new framework and encourage its practice. Integrating China into the international toxicity testing and regulatory framework would demonstrate the material benefits of global harmonization. In many aspects, China presents an intriguing case as it begins to respond to the need for developing and utilizing alternative approaches in toxicity testing. China is currently at the crossroads; the opportunity to play a significant role in the development of alternative testing strategies and participation is here and now, not at some time in the future.
